# Intrathecal CAR-NK cells infusion for isolated CNS relapse after allogeneic stem cell transplantation: case report

**DOI:** 10.1186/s13287-023-03272-0

**Published:** 2023-03-20

**Authors:** Jing Yuan, Fuxu Wang, Hanyun Ren

**Affiliations:** 1 Department of Hematology, The Second Hospital of Hebei Medical University, 215 Heping West Road, Shijiazhuang, 050000 Hebei China; 2 Department of Hematology, Peking University First Hospital, 8 Xishiku Street, Xicheng District, Beijing, 100034 China

**Keywords:** CAR-NK cells, Intrathecal infusion, CNS relapse, Allogeneic stem cell transplantation, Case report

## Abstract

A 24-year-old man with central nervous system (CNS) involvement of T-cell lineage acute lymphoblastic leukemia received sibling allogeneic stem cell transplantation (allo-SCT). He developed isolated CNS relapse early post-SCT, while high-dose systemic chemotherapy, intrathecal (IT) triple infusion and IT donor lymphocytes infusion (DLI) all demonstrated effectiveness. We performed IT umbilical cord blood-derived CAR-NK (target CD7) cells infusion, which was not previously reported. After infusion, detection of cytokines revealed that interferon-γ, interleukin-6 and interleukin-8 increased in CSF. He developed high fever, headache, nausea, vomiting and a spinal cord transection with incontinence in a short time, whereas the ptosis and blurred vision improved completely. The bone marrow remained encouragingly complete remission and complete donor chimerism over 9 months after IT CAR-NK cells infusion. In conclusion, IT CAR-NK cells infusion is a potentially feasible and effective option for patients with CNS relapse, with limited neurological toxicity.

## Introduction

Central nervous system (CNS) relapse is a major obstacle for patients with hematologic malignancies after allogeneic stem cell transplantation (allo-SCT). The incidence is 2 ~ 5.5% among patients without previous CNS involvement and up to 11 ~ 27% with previous CNS involvement [1]. Intrathecal (IT) injection, cranial irradiation, high-dose chemotherapy, and tandem SCT are general options for CNS relapse, but intensive chemotherapy is inappropriate for patients with transplantation related complications post-SCT. In addition, these treatments always come with acute or long-term neurotoxicity [2–5]. Leukemia relapse in the CNS after conventional therapy is difficult to treat and is associate with poor prognosis [6].

Although chimeric antigen receptor (CAR) T cell therapy has achieved remarkable success in the treatment of leukemia, there remain a lot of deficiencies in CAR-T cell therapy, particularly the cytokine release syndrome (CRS). CAR-engineered natural killer (CAR-NK) cell therapy is expected to remedy some of these deficiencies. Herein, we present a patient with T-cell lineage acute lymphoblastic leukemia (T-ALL) who underwent IT CAR-NK cells derived-from umbilical cord blood (UCB) infusion for isolated CNS relapse early post-SCT.

## Case presentation

**Fig. 1:**
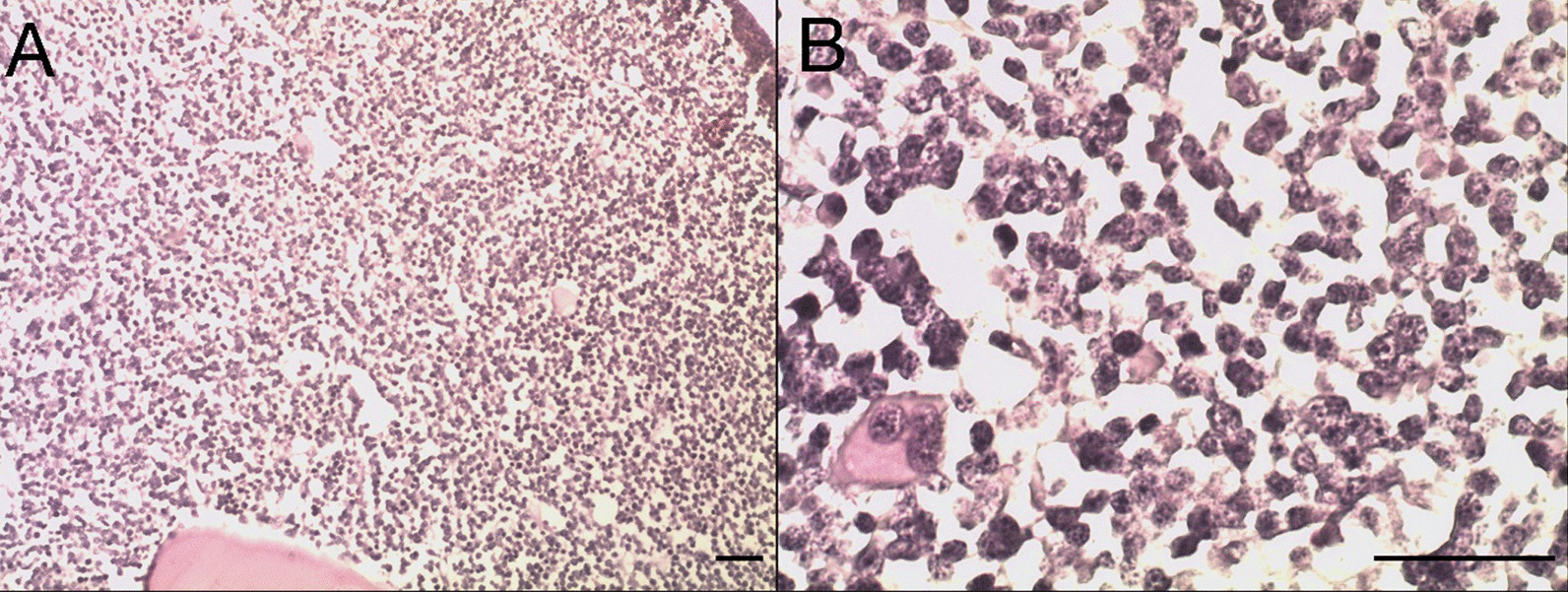
**A** When visualized at a low magnification (magnification, objective × eyepiece, 10 × 10), the BM biopsy revealed large aggregates of blasts. **B** BM biopsy demonstrating a predominance of lymphoblasts (40 × 10). Scale bar, 100 μm for (**A**, **B**). The images were observed with microscope (OLYMPUS BX41, Japan) and acquired by the Pathological Quality Control System (JEDA, China)

A young man of 24 years old was diagnosed with cortical T-ALL. He had been healthy with no history of medical disorders or surgical procedures. His family history was unremarkable for metabolic diseases or any other hereditary conditions. The initial laboratory findings showed white blood cells count was 2,110/µL, while bone marrow (BM) was with 89% of lymphoblasts (Fig. 1). The leukemia cells were positive for CD2, CD3, CD4, CD8, CD7, CD1α and cytoplasmic CD3, but negative for B-cell and myeloid cell markers with flow cytometry (FCM) analysis. The results were analyzed with CellQuest software (BD, Mountain View, CA, USA). Chromosomal karyotyping revealed a normal karyotype of 46, XY, while T-cell receptor (TCR) rearrangement confirmed by polymerase chain reaction revealed T-cell clonality. The brain CT scan revealed negative, while lumbar puncture test showed no increased cells or protein level in cerebrospinal fluid (CSF). All tests above ruled out initial CNS involvement. After induction chemotherapy regimen, initial response evaluation achieved complete remission. He then received two cycles of consolidation regimen, while FCM monitoring for minimal residual disease (MRD) revealed 1.92% lymphoblasts [7, 8]. MRD assessment remained persistently positive, so the patient intended to receive peripheral blood SCT with his elder sister as a human leukocyte antigen (HLA) identical sibling donor.

However, 10 days after a high-dose intravenous methotrexate and cytarabine regimen before SCT, he developed a sudden onset of blepharoptosis of right eye with left deviation of mouth [9]. These signs lasted for seconds each time. There were no positive findings on magnetic resonance imaging (MRI) and computed tomography scans. Diagnostic lumbar spinal puncture was performed immediately. The cells count in CSF was 2/µL, with no leukemic cells observed. IT triple therapeutic infusion of cytarabine 30 mg, methotrexate 10 mg, and dexamethasone 5 mg was administered concomitantly at the diagnostic lumbar puncture. The symptoms were shortly relieved. On basis of neurological symptoms and therapeutic response, diagnosis of CNS relapse was made. Subsequently he received systemic chemotherapy of high-dose methotrexate plus IT triple therapeutic infusions twice weekly. The neurological symptoms were obviously relieved. The BM FCM-MRD revealed down to 0.25%.

The conditioning regimen comprised whole brain and total spinal cord radiotherapy, venetoclax, hydroxycarbamide, cytarabine, busulfan, cyclophosphamide and Methyl-CCNU. The prophylaxis of graft-versus-host disease (GVHD) included mycophenolate mofetil, cyclosporin, and short-term methotrexate. The engraftment was confirmed without complications. He developed no acute or chronic GVHD. The FCM monitoring for MRD achieved persistent negative remission, with complete donor chimerism in BM and peripheral blood.

**Fig. 2:**
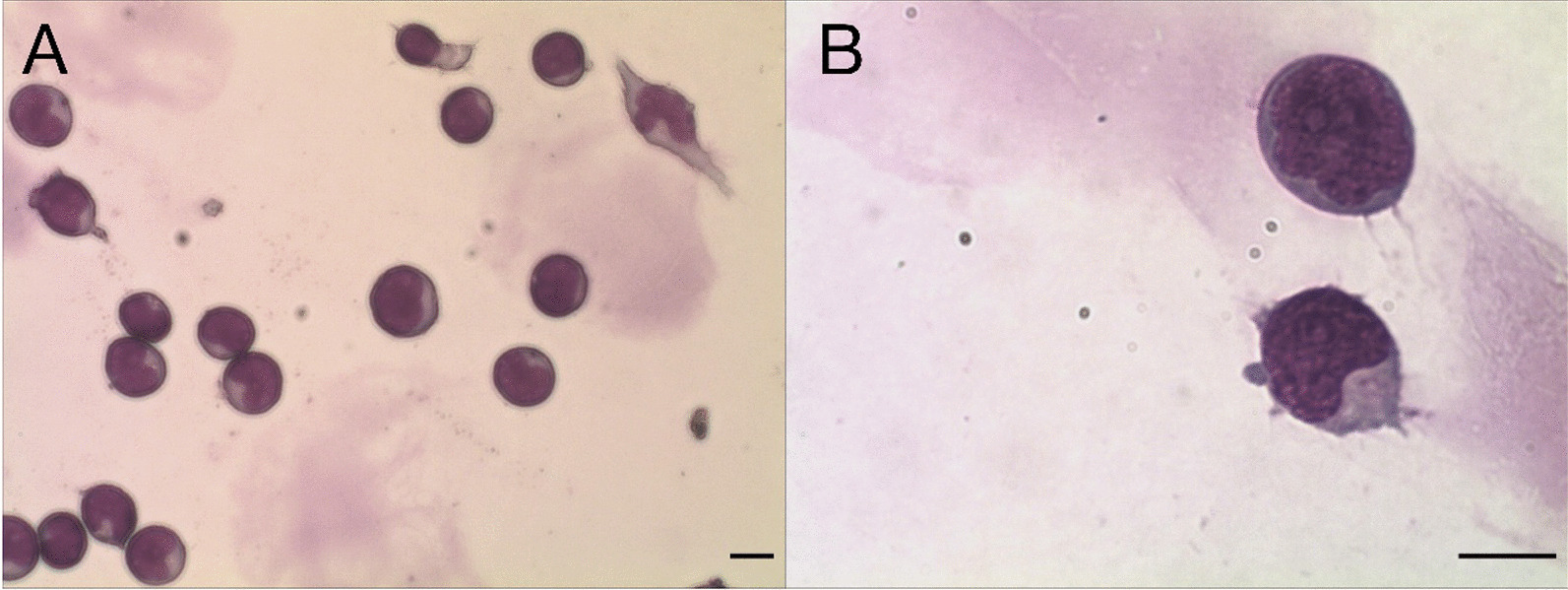
The CSF exfoliative cytology after centrifugation. **A** When visualized at a low magnification (40 × 10), lots of leukemia blasts were observed. **B** The lymphoblasts were easily to be seen (100 × 10). Scale bar, 10 μm for (**A**, **B**)

On day 83 post-SCT, his left eye developed esophoria with limited abduction and double vision, which was similar to previous manifestations. Lumbar spinal puncture showed increased CSF pressure and cell counts. The exfoliative cytology showed that leukemic blasts were easily to be seen (Fig. 2), while FCM analysis revealed 87% of lymphoblasts. After cycles of high-dose methotrexate plus repeated IT triple therapy twice weekly, cell counts in CSF decreased to 2/µL. Meanwhile his neurological signs improved. During this process, the MRD monitoring through FCM revealed persistently negative. No lymphoblasts were found with complete donor chimerism both in BM and peripheral blood.

His neurological symptoms got worse again 4 weeks later. The patient developed progressive paraplegia without urine and feces incontinence. To treat myelitis, the patient received intravenous immunoglobulin and methylprednisolone. However, the neurological signs were not obviously improved. He had already received a therapeutic radiotherapy and many cycles of systemic chemotherapy with IT injection. We therefore performed IT DLI in dose of 1.23 × 10^9^/L. But IT DLI had limited efficacy and his neurological signs seemed no obvious improvement. In the meantime, he developed progressive legs numbness or weakness and activity obstacle.

On day 131, we performed IT umbilical cord blood-derived CAR-NK cells (target CD7) infusion (total volume 3 ml, with NK cells count 1.0 × 10^7^). The donor lymphocytes and CAR-NK cells were prepared using a local distributed, standardized, automated system at the cell processing center of Hebei Senlang Biotechnology Company under good manufacturing practice conditions. After IT CAR-NK infusion, he developed high fever, headache, nausea and vomiting. Three days later, he developed a spinal cord transection with incontinence. Physical examination revealed absence of touch and pain sensation below the level of the third lumbar vertebra. Muscle strengths of lower extremities declined to class zero [9]. MRI of brain and spinal cord revealed subacute combined degeneration. Detection of cytokines revealed that interferon-γ, interleukin-6 and interleukin-8 (R&D Systems, Minneapolis, MN, USA) increased in CSF. After IT CAR-NK cells infusion, his limb numbness and movement disorder got worse in a short time, whereas the ptosis and blurred vision improved completely. BM and blood simultaneously remained CR and complete donor chimerism over nine months after IT CAR-NK cells infusion.

## Discussion

The majority of patients with CNS relapse post-SCT usually coincide with or predict soon afterwards for systemic relapse. Univariate analysis indicated that patients with CNS involvement had worse survival after allo-SCT due to a higher relapse incidence. Cranial irradiation is still the most effective treatment for overt CNS leukemia, but previous studies have demonstrated that neither approach successfully decreases CNS recurrence. Our T-ALL patient with MRD positive and CNS involvement before SCT predicted a higher rate of relapse. He had undergone cycles of high-dose systemic chemotherapy, IT triple therapy and IT DLI after CNS recurrence. But the neurological signs only relieved for about 4 weeks. The efficacy of IT DLI was probably insufficient for CNS relapse, because relapsed leukemia cells in CNS have already escaped from circulating donor T cells. Regrettably, he developed progressive inflammation of the brain and spinal cord. A history of cranial irradiation before SCT was considered as a probable risk factor for CNS complications. It suggested us to find a more effective therapy.

Adoptive immunotherapy based on NK cells has shown clinical benefits in patients of leukemia [10–12]. Since CNS was considered to be an immunologic sanctuary protecting lymphoblasts from NK-cell activity, we planned IT CAR-NK cells infusion. In present case, we performed IT CAR-NK cells derived from unrelated UCB infusion. After IT infusion, his neural symptoms significantly worsen. We speculated the causes were various. First, the patient had received cranial irradiation, multiple high-doses of intravenous chemotherapy plus IT therapy. Serious and unexpected neurotoxicity was observed and progressive aggravation. Second, the proinflammatory cytokines levels in CSF increased. IT CAR-NK infusion might cause CRS, which led to neurological complications. It suggested us that IT CAR-NK infusion was not completely safe, with potential neurological toxicity. This may, in part, be improved by decreasing the CAR-NK cells dose or increasing the sessions of IT infusion.

Nevertheless, his initial signs with limited abduction and double vision significantly improved for more than 8 weeks after CNS recurrence, while BM and blood maintained in CR and complete donor chimerism. IT CAR-NK infusion limited the outgrowth of leukemia cells in the periphery. We hypothesized that the direct infusion of CAR-NK cells into CSF can provoke an appropriate graft-versus-leukemia effect both on marrow and blood. Hence, prophylaxis of CNS relapse may be needed with emerging NK cell-based therapies against leukemia. Previous studies demonstrated that activated NK cells by IL-15 or other cytokines inhibit systemic peripheral leukemia but cannot enter CNS to control the leukemia relapse [13–15]. IL-15 is essential to the development, survival and activation of NK cells [16].

### Patient perspective

Finding a novel therapy for CNSL is important and meaningful. Though the patient exhibited CNS events after IT CAR-NK cells infusion, he was aware that this could be possible adverse events of the immunotherapy, or a prolonged sequelae of radiation and combination chemotherapy. The patient has kindly submitted his own account of events until loss to follow-up. There were encouraging results that he remained a state of BM CR and complete donor chimerism over nine months after IT CAR-NK cells infusion.

## Conclusions

IT CAR-NK cells infusion is a potentially feasible and effective option for patients with CNS relapse, with limited neurological toxicity. However, it is necessary to improve the efficacy by modifying current method, such as the initial CAR-NK cells dose adjustment, increasing the sessions of IT infusion and improving the CAR-NK cells preparation process.

## Data Availability

The datasets used or analyzed during the current study are available from the corresponding author on reasonable request.

## Abbreviations

CNS: Central nervous system
IT: Intrathecal
CAR-NK: Chimeric antigen receptor-engineered natural killer
allo-SCT: Allogeneic stem cell transplantation
DLI: Donor lymphocytes infusion
CRS: Cytokine release syndrome
T-ALL: T-cell lineage acute lymphoblastic leukemia
UCB: Umbilical cord blood
BM: Bone marrow
FCM: Flow cytometry
TCR: T-cell receptor
CSF: Cerebrospinal fluid
MRD: Minimal residual disease
HLA: Human leukocyte antigen
GVHD: Graft-versus-host disease
